# Ecotoxicity of a Representative Urban Mixture of Rare Earth Elements to *Hydra vulgaris*

**DOI:** 10.3390/toxics12120904

**Published:** 2024-12-12

**Authors:** Joelle Auclair, Chantale André, Eva Roubeau-Dumont, François Gagné

**Affiliations:** Aquatic Contaminants Research Division, Environment and Climate Change Canada, 105 McGill, Montréal, QC H2Y 2E7, Canada; joelle.auclair@ec.gc.ca (J.A.); chantale.andre@ec.gc.ca (C.A.); eva.roubeaudumont@ec.gc.ca (E.R.-D.)

**Keywords:** *Hydra vulgaris*, oxidative stress, protein-recycling autophagy, regeneration, DNA damage

## Abstract

Rare earth elements (REEs) are considered as emerging contaminants due to their use in the fabrication process of current technologies. As such, their aquatic toxicity, especially as a mixture, is not well understood, as it has been scarcely investigated. The purpose of this study was to shed light on the sublethal and lethal toxicity of a realistic mixture of five REE in *Hydra vulgaris*. The REE mixture was composed of five elements (Gd, Ce, Nd, Y and Dy, with a total REE concentration of 0.137 µg/L = 1× concentration) that were found in six municipal effluents in Canada at the same concentration ratios. The organisms were exposed to increasing concentrations (0.5, 1, 5, 10, 25, 50 and 100×) of the mixture for 96 h at 20 °C. The lethal and sublethal toxicities were evaluated by morphological changes and the gene expression (mRNA) involved in oxidative stress, damaged protein salvaging (autophagy for the reabsorption of damaged proteins), regeneration, neural activity and DNA repair of oxidatively damaged DNA. The data revealed that the total REE concentration of the environmental mixture was well below the lethal concentrations of the individual REEs, which occur generally at concentrations > 200 µg/L. This study proposes a novel gene transcription set to investigate the mode of action where gene expression changes occurred at concentrations below those reported in municipal effluents, suggesting long-term toxic effects in hydras close to municipal effluent discharges. This suggests that the release of REEs by municipal/hospital (for Gd) effluents should be more closely monitored.

## 1. Introduction

Rare earth elements (REEs) represent an important family of critical elements of technology. REEs are used for various electronic components such as semiconductors, plasma screens, LED and batteries [1,2]. As a consequence, REEs are extracted at large scales over the world, reaching some 300,000 tons in 2022 to support technological development [3]. REEs from various electronic dsevices are ultimately discarded in solid disposal sites, making these elements contaminants of emerging concern [4,5].

The relative composition of REEs found in mine-contaminated lakes differs from that in municipal wastewater draining solid waste disposal sites [6,7]. For example, the major REEs from 10 mining contaminated lakes consist of La, Ce, Pr, Nd and Sm, with a total loading of 580 µg/L at concentration ratios similar to those found in the Earth’s crust [8]. These REEs are operationally called mining REE mixture and re-enter the environment through the disposal of consumer and industrial products from landfills and wastewater discharged from both domestic and industrial processes [8]. However, the major REEs in municipal effluents occur at much lower concentrations and differ in composition from the mining polluted lakes. The five major REEs (found at levels > 1 ng/L) from six municipal effluents in Canada, based on Turcotte et al. [7], consist of gadolinium (Gd), cerium (Ce), neodymium (Nd), ytterbium (Y) and dysprosium (Dy), at concentration ratios differing from the Earth crust composition, with a mean total REE concentration of 0.137 µg/L. These REEs are operationally termed as urban REE mixture, and characterized by the Gd anomaly [9,10]. Gd is used as contrast agent during magnetic resonance imaging and is released mostly in the dissolved phase (urine elimination) in hospitals and wastewater treatment plants [6,11,12]. It was shown that one hospital effluent released about 40–50 ng/L of Gd, where the secondary treated effluent released 26 ng/L Gd. The removal efficiency of Gd by municipal effluents oscillates between 20 and 40%, suggesting a significant release into aquatic ecosystems [7]. However, the toxicity of the urban mix has yet to be examined to determine whether the urban REE mixture poses a similar toxic risk to hydras at realistic environmental concentrations.

*Hydra vulgaris* Pallas, 1766, belongs to the Hydrozoa class of the Cnidaria phylum and is found in freshwaters. This organism has been cultivated in the laboratory since the 1950s [13] and was more recently used in bioassay to investigate the toxicity of various xenobiotics and liquid mixtures [14,15]. Hydras are relatively small organisms (between 2 and 10 mm in length) with a tubular body and a head composed of seven tentacles (Figure S1). They feed on small prey, such as copepods and other zooplankton. They have unique regenerative abilities, growing and reproducing without aging [16,17]. Hydras are simple organisms composed of two layers of epithelial cells, making them very sensitive to various contaminants. An attractive feature of the hydra bioassay is that the intensity in toxicity can be visually observed by characteristic morphology alterations (Figure S1). First, tentacles form a button/bud at the tip and retract, followed by severe tentacle contraction at the body, forming a tulip-like appearance, followed by disintegration of the body. Tentacle budding and retraction are considered reversible (sublethal) changes as they regenerate when the stressor is removed from the media. Severe tentacles formation, like tulip stage and body disintegration, are considered irreversible (lethal), exceeding the organism’s ability to regenerate. Hydras are considered a sensitive test species for ecotoxicity testing [18,19], with its sensitivity surpassing that of the rainbow trout toxicity test, most notably for metals and rare earth elements [20]. The small size of these organisms complicates investigations at the biomolecular level, requiring an important amount of starting material or highly sensitive technologies. Reverse transcriptase polymerase chain reaction (RT-PCR) represents a very sensitive methodology for quantifying specific mRNA targets. A novel quantitative RT-PCR methodology was developed to determine the effects of xenobiotics on the gene expression involved in oxidative stress, oxidated DNA (8-oxo-guanosine) repair, protein salvaging and tagging by the ubiquitin–proteasome pathway, autophagy, cell regeneration and neural activity. The analysis of gene expression changes preceding changes in morphology could be of predictive value in preventing toxicity and improving understanding of the mode of action of environmental contaminants. A previous study examined the toxicity of the mining REE mixture (at the same relative concentration found in contaminated lakes) in *Hydra attenuata* and revealed antagonistic interactions between the REEs based on the individual toxicity profiles of each REE [6]. Nevertheless, the mining mixture reduced reproduction, head regeneration and irreversible morphology (mortality) at concentrations below those reported in contaminated lakes.

This study aims to evaluate the sublethal and lethal effects of urban REE mixtures on Hydra vulgaris at morphological and molecular levels to identify thresholds of toxicity and understand their mechanisms. The sublethal toxicity was also examined at the molecular level to better understand the mode of action of REE mixtures that precedes morphological alterations. An attempt was made to determine the threshold concentrations of molecular changes that occur before the onset of altered morphology in hydras.

## 2. Methods

### 2.1. Sample Preparation

Pure powders of the REE (Gd(III), Ce(III), Nd(III), Yb (III) and Dy(III) as trichloride salts) were purchased from Sigma-Aldrich (Mississauga, ON, Canada). They were prepared in the following proportions based on the reported concentrations (dissolved phase) found in Canada’s wastewater effluents [7]: Gd (110 ng/L), Ce (9 ng/L), Nd (8 ng/L), Yb (6 ng/L) and Dy (4 ng/L) corresponding to a total REE loading of 137 ng/L. This mixture is referred to as the urban mixture where the 1× mixture represents the actual concentrations of REE in the dissolved phase in municipal effluents. These concentrations are relatively low and far below their water solubility (>1 g/100 mL), where precipitation is not expected. The conductivity of the 1000 and 500× solutions was measured following 1 and 96b h dissolution in MilliQ water or the Hydra medium and revealed no loss of ion activity.

### 2.2. Aquatic Toxicity Assessment with Hydra vulgaris

*Hydra vulgaris* were reared in 100 mL crystallization bowls with the Hydra medium: 1 mM CaCl_2_ containing 0.4 mM TES buffer pH 7.5 without EDTA [16]. They were fed daily with live *Artemia salina* brine shrimps as previously described. They were allowed to grow and reproduce under 16 h/8 h light/dark cycles at 20–22 °C with a doubling time of 4.5 days. Hydras were not fed prior to the initiation of the exposure experiments. Adult hydras (3) were placed in each of three wells in 24-well microplates with 4 mL of freshly prepared Hydra medium. They were exposed to increasing concentrations of the REE urban mixture at a concentration range of 0.5, 1, 10, 25, 50 and 100× for 96 h at 20 °C. This represents a total REE range of 0.0685 to 13.7 µg/L. The selection of 96 h as the exposure time was based on the time needed for the appearance of morphological changes, which requires at least 72 h to manifest [21]. The hydras were randomly selected and not in reproduction mode (characterized by polyp formation or budding, Figure S1). For trancriptomics analysis, 2 other microplates were prepared. The lethal and sublethal toxicities were determined and expressed as the lethal concentration of 50% of the hydras (LC50) and the sublethal effect concentration of 50% of the hydras (EC50) using the Spearman–Karber method [22]. Given that the exposure period encompassed the doubling time, the exposure period was considered chronic. The morphological changes for lethality (tulip and disintegrated body) and sublethality (budding and shortening of tentacles) were determined using a 6× stereomicroscope. Hydras with unaffected morphologies were collected for gene expression analysis at the concerned exposure concentrations. Hydras were rinsed in Hydra media and harvested with a 1 mL pipet to be immediately transferred in RNA later solution (Millipore Sigma, Burlington, ON, Canada) and stored at −20 °C for gene expression analysis.

### 2.3. Gene Expression Analysis

Preliminary experiments revealed that RNA extraction from 2 pooled wells (N = 6 total hydras per treatment) were sufficient for RNA purity analysis. Hence, gene expression analysis was performed with 3 groups of 2 wells. Total RNA was extracted from each pooled hydra using the RNeasy Plus Mini Kit (Qiagen, Montreal, QC, Canada). RNA concentration and purity were assessed with the NanoDrop 1000 (Thermo Fisher Scientific, Montreal, QC, Canada) and RNA integrity was confirmed using the TapeStation 4150 system (Agilent) with the Agilent RNA ScreenTape Assay (cat # 5067–5576, Agilent Technologies Inc., Santa Clara, CA, USA). Reverse transcription was performed with the QuantiTect^®^ Reverse Transcription Kit (Qiagen), ensuring the complete removal of genomic DNA. The resulting cDNA samples were stored at −80 °C until quantitative real-time PCR (qPCR) analysis.

For each target gene (Table 1), the selected forward and reverse primers were validated with a cDNA concentration of 10 ng, followed by 6–8 serial dilutions (10, 8, 6 ng, etc.) with an amplification performance between 95% and 115. This allowed us to establish the limit of quantification for each target gene. Each reaction was run in duplicate and consisted of 5 µL cDNA, 6.5 µL of 2× SsoFast EvaGreen Supermix (Bio-Rad, Montreal, QC, Canada), 300 nM of each primer and DEPC-treated water (Ambion, Montreal, QC, Canada) up to a total volume of 13 µL. The cycling parameters were as follows: 95 °C for 30 s, and then 40 cycles of 95 °C for 5 s and 60 °C for 10 s for HPRT (reference gene), RPLPO (reference gene), Efα (reference gene), DDC1, SRF and OGG; 95 °C for 30 s, and then 40 cycles of 95 °C for 5 s and 56 °C for 10 s for CAT and MANF; and 95 °C for 30 s, and then 40 cycles of 95 °C for 5 s and 56 °C for 30 s for MAPC3. All qPCR analyses were conducted using SsoFast^TM^ EvaGreen^®^ Supermix (Bio-Rad, Mississauga, ON, Canada) and the CFX96 Touch Real-Time PCR Detection System (Bio-Rad, Mississauga, ON, Canada). Amplification specificity was verified by denaturation (melting curve) temperature analysis at the end of the amplification cycles. A no-template control (NTC) was included on each plate. Data analysis was performed using CFX Maestro (Bio-Rad).

### 2.4. Data Analysis

The exposure experiments were repeated twice with n = 3 replicates for each treatment. Toxicogenomic data were expressed as effect threshold concentrations (X or µg/L total REE concentrations) and defined as follows: effect threshold = (no effect concentration × lowest significant effect concentration)^1/2^. The gene expression data were analyzed using a rank-based analysis of variance followed by the Conover–Iman test for differences from the controls. Relationships between toxicity and the gene expression data were determined using the Spearman rank procedure. The gene expression data were also analyzed by hierarchical tree to determine similarity of effects between the elements using the square Pearson-moment correlation (1-R) as the metric distance between the observed gene expression changes. Significance was set at *p* < 0.05. All the statistical analyses were conducted using SYSTAT (version 13, San Jose, CA, USA).

## 3. Results and Discussion

The physical–chemical properties of the REEs in the urban mixture are provided (Table 2). The mixture was composed with the following proportion of REEs: Gd (80%) < Ce (7%) < Nd (6%) < Yb (4%) < Dy (3%), with a total REEs mass concentration of 0.137 µg/L for the 1× treatment. Both the levels and relative proportion of REEs in this mixture were calculated based the dissolved REE levels from six different municipal effluents in Canada [7]. The atomic mass range was relatively narrow, between 140.12 and 173.04 g/mol, with an ionic radius range between 91 and 107 pm (Table 2). Electronegativity generally increased with the atomic mass and, to some extent, with the ionic radius. These metrics were relatively far from the major trivalent bioelement in organisms, Fe (atomic weight 55.85, ionic radius 79 pm and electronegativity of 1.83) [23]. The lethal and sublethal toxicity data for each individual REE in the hydras are also reported in Table 3. In general, lighter REEs with higher ionic radii were more toxic to hydras, corroborating previous findings on REEs LC50 and EC50 values in hydras [6]. The lethal (LC50) and sublethal (EC50) concentrations ranged from 310–690 µg/L to 50–270 µg/L, respectively, based on morphological changes (Table 3). This suggests that the 100× urban mixture for Gd (11 µg/L) was 47 times less concentrated than the LC50 for Gd and 9 times lower than the EC50 for Gd in hydras. The highest reported levels of dissolved Gd in municipal wastewaters reached 229 ng/L in the dissolved fraction for a secondary activated sludge effluent in the Saint Lawrence River area [7]. In another study, dissolved Gd levels reached 286 ng/L at an advanced wastewater treatment plant on the American east coast (Virginia, USA) [24]. These values could reach higher concentrations (700 µg/L) in highly populated areas (37 million inhabitants) with many hospitals, since Gd used as a contrast agent in MRI represents the main source of dissolved Gd in effluents [25].

**Figure 1:**
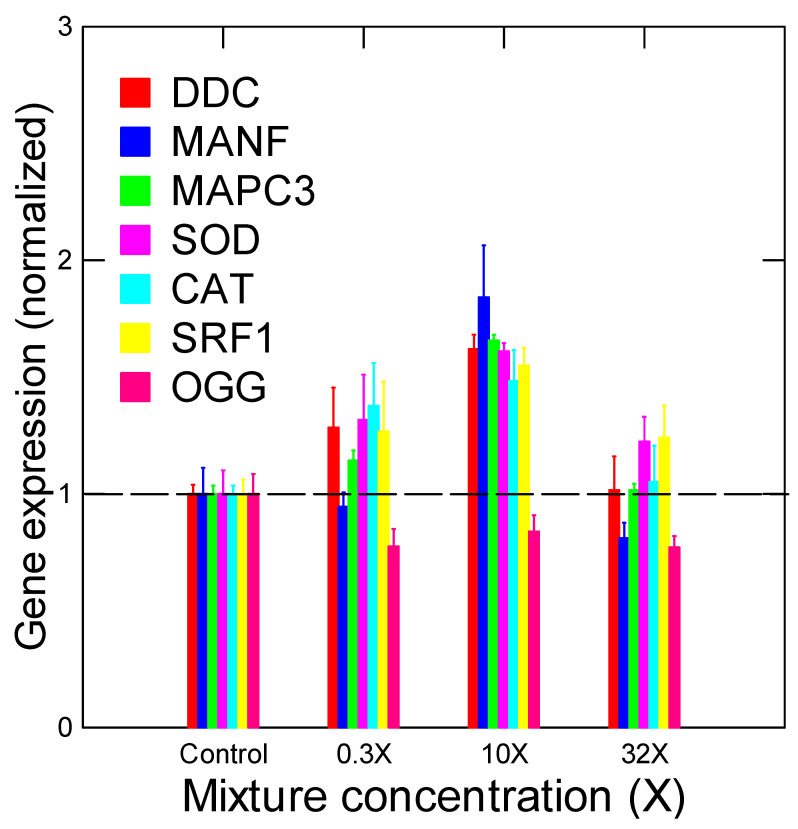
Gene expression changes in hydras exposed to the REE mix. The data represents the mean with the standard error. The 1× concentration consists of Gd (0.11 µg/L), Ce (0.009 µg/L), Nd (0.008 µg/L), Yb (0.006 µg/L) and Dy (0.004 µg/L), giving a total REE loading of 0.137 µg/L.

The sublethal effects of the representative mixture were investigated in *Hydra attenuate,* as shown in Table 3. The urban mixture did not lead to sublethal morphological changes in hydras up to an equivalent of 13.7 µg/L of total REEs, where Gd represented 80% of the total mass. However, significant changes were observed in the mixture for all the examined gene targets (Figure 1). Most genes were upregulated at mixture concentrations between 0.5× (0.0685 µg/L total REE) and 25× (3.25 µg/L total REE), except for the downregulated expression of OGG gene involved in the repair of oxidatively damaged DNA. The responses were dampened at the highest concentration tested of 100× (13.7 µg/L total REE), indicating “pre-morphological toxicity” given that the Gd (11 µg/L) in the urban mixture was approaching the EC50 range of Gd (first appearance of tentacle budding at 40 µg/L for Gd). This indicates that municipal effluents release Gd at concentrations that could induce changes in gene expression in hydras, especially for those involved in oxidative stress (CAT and OGG) and protein salvage pathways (MAPC3), which precede sublethal morphological changes (Table 3). To the best of our knowledge, this is the first study that uses hydra transcriptomics for the investigation of the mode of action at the fundamental/molecular level. Most transcriptomics studies of hydras involve gene analysis for the normal functioning of this species [26]. A whole transcriptome analysis was performed on hydras, leading to circa 32 K accessible (downloadable) annotated transcripts. Most reported studies using transcriptomics involved the mechanism of head regeneration, growth factors in the ectoderm, neurogenesis, aging and circadian rhythms with clock genes [27,28,29,30]. We found two studies dealing with the toxicity of quantum dots (nanoparticles) in hydras [31,32]. Gene expression changes were found for heat shock proteins, peroxidase, ubiquitin conjugated proteins and protein synthesis. Future research should examine longer exposure periods (>96 h) in hydras for REEs including molecular biomarkers. In zebra mussels exposed to either GdCl_3_ or the medical organic form Gd used for medical imaging (Omniscan), CAT and SOD gene expression were decreased at concentrations ranging from 10 to 50 µg/L, followed by significant increases at a much higher concentration (1250 µg/L) [33]. Decreased OGG gene expression suggests an accumulation of DNA damage (8-oxo-guanosine adducts), which could lead to cytogenetic damage. Indeed, Gd was reported to increase the frequency of micronuclei in human lymphocytes [34,35] and in the plant *Arabidopsis thaliana* at environmentally relevant concentrations in soils [36].

**Figure 2:**
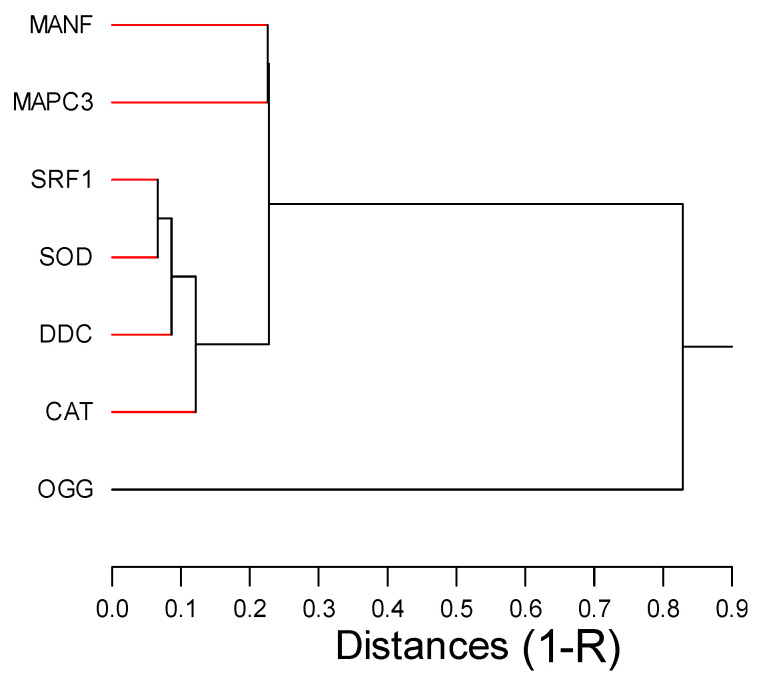
Hierarchical tree analysis of gene expression data. The relative distance between gene targets was computed by the square correlation coefficient (1-R). The red bars indicate significant trends (*p* < 0.05).

Correlation analysis revealed that SRF-1 gene expression was clustered (i.e., strongly correlated) with oxidative stress (SOD and CAT) and DDC biomarkers (Figure 2). DDC activity increases the conversion of L-DOPA to dopamine during the wake stage, feeding activity and tentacles regeneration [37,38]. This is consistent with the significant correlation between DDC and SRF1 gene expression involved in regeneration and cell differentiation. This suggests that genes involved in oxygen radical elimination (CAT and SOD) are coupled to hydra neural activity (DDC) and cell regeneration. In a previous study, hydras were exposed to a representative mixture of REE from lakes contaminated with mine tailings, which led to decreased head (tentacles) regeneration and reproduction rates [6]. The mixture consisted of the five most abundant REEs (La, Ce, Pr, Nd and Sm), two of which are also found in the urban mixture at a total REE concentration of 580 µg/L, which is 42 times more concentrated than the urban effluent mixture. Hence, mining mixtures present a higher risk in hydras compared to the urban mixture. Nevertheless, the threshold effect concentrations for lethal and sublethal toxicities were between 0.3 and 0.7 µg/L of the total REE loading, which is in the same order of magnitude as the present study (<0.0685–0.137 µg/L total REEs loading) for gene expression changes. Furthermore, hydra doubling time was significantly reduced at 0.2× (corresponding to 116 µg/L total REE loadings) in the mining lake mixture, indicating that hydras could be used as a sensitive model organism for the assessment of aquatic ecotoxicological risks of REE in both mining contaminated lakes and urban mixtures. More research on early biochemical and/or gene expression levels as well as longer exposure time should improve our understanding of the long-term effects of REE mixtures in aquatic environments.

In conclusion, hydras exposed to increasing concentrations of a realistic urban mixture did not lead to morphological changes, but significant changes at the gene expression level for protein salvaging and oxidative stress occurred at concentrations below those found in municipal effluents. Furthermore, genes involved in neural activity, regeneration and oxidative stress were upregulated at concentrations 3.5 times higher than those found in the effluents, but these concentrations could be reached in urban effluents from more-populated cities.

## Figures and Tables

**Table 1:** Gene transcript identity and sequences.

Function	Gene Name	Forward/Reverse (5’---->3’)	Amplicon (bp)
*Housekeeping genes*	*Hypoxanthine-guanine phosphoribosyltransferase-like* *HPRT*	*GAA TTG AAC GCA TGG CTC GT/* *GTC TTG GCT GAA CCG AAA ACC*	*98*
	*60S acidic ribosomal protein P0-like* *Rplp0-1*	*CTG AGG CTG CTC TTC TTG CT/* *GGA CTG AAA ATG CTT CCG TTG T*	*94*
*Autophagy and Ub-pathway*	*(Microtubule-associated protein 1 chain 3 light)* *MAPC3l*	*CCA GAG AAA GCG AGA ATC CGA/* *TGG AGA GCA TAC CAA CTG TCA T*	*152*
	*Mesencephalic astrocyte-derived neurotrophic factor homolog * *MANF*	*CCA CTC GCA TAC TAC AAG CCT/* *ACA ACC ACT ACA AGT CTC ACC C*	*180*
*Stress and antiox*	*Superoxide dismutase [Cu-Zn]-like * *SOD*	*ACC TGG TAA GCA CGG TTT TCA/* *TGC ACC ACT CCA TCT TTA CCA*	*171*
	*Catalase-like* *CAT*	*ACA GCC TCA ATG ACT GTT GGG/* *CCA CTC CAT TCA GAG CAG CC*	*196*
*DNA damage and repair*	*8-Oxoguanine DNA Glycosylase* *OGG*	*TGT GAC TGG AGT TGA AGA TGC T/* *ACT CCA GGC AAT GAG CAA AGA*	*174*
*Regeneration and stem factor*	*Serum Response Factor* *SRF1*	*CTT GTG GCA TCG GAA ACA GG/* *TGC TTT GCC ACT TTC AGA GGT A*	*84*
*Neural activity*	*Dopa Decarboxylase* *DDC*	*GCC CCA GTT GAG CCA GAT AA/ CAG TGA GTG ACA CCT GGC AT*	*77*
*Protein synthesis*	*Elongation factor-1 alpha* *EL1*	*TGC TCC TGG ACA TCG TGA CT/* *CAA CGA TGA GTA CGG CAC AAT C*	*77*

**Table 2:** Physico-chemical characteristics and toxicity data of individual rare earth elements in mixture.

REE	1× Mixture µg/L	Atomic Mass	Electronegativity	Ionic Radius (pm)	Individual LC50 Hydra µg/L (95% CI) ^1^	Individual EC50 Hydra µg/L (95% CI) ^1^
Gd	0.11	157.25	1.2	97	520 (430–630)	100 (70–150)
Ce	0.009	140.12	1.12	107	330 (240–450)	50 (30–70)
Nd	0.008	144.24	1.14	104	310 (250–390)	90 (60–130)
Yb	0.006	173.04	1.19	102	505 (304–705) ^2^	128 (88–170) ^2^
Dy	0.004	162.5	1.22	91	690 (560–840)	270 (220–320)
	Total: 0.137 µg/L					

^1^. From [13]. ^2^. From the regression model between LC50/EC50 with the atomic weight and ionic radius: Hydra LC50 (µg/L) = 1613 − 17 (ionic radius) + 3.6 (atomic mass); R = 0.96, *p* = 0.003 and HydraEC50 (µg/L) = 915 − 9.3 (ionic radius) + 0.93 (atomic mass), R = 0.74, *p* = 0.05.

**Table 3:** Toxicity data of REE mixture.

Gene Targets	DDC	MANF	MAPC3	SOD	CAT	SRF1	OGG	Hydra LC50	Hydra EC50
Effects Threshold (X)	3.5 X	3.5 X	<0.5 X	3.5 X	<0.5 X	3.5 X	<0.5 X (inhibition)	>100 X	>100 X
Effects Threshold (total REE µg/L)	0.48	0.48	<0.0685	0.48	<0.0685	0.48	<0.0685	>13.7	>13.7

## Data Availability

The data will be available upon request to the authors.

## Supplementary Materials

The following supporting information can be downloaded at: https://www.mdpi.com/article/10.3390/toxics12120904/s1. Figure S1: Characteristic morphological changes in hydras during toxicity.
