# Supplementary material for: Ecotoxicity of a Representative Urban Mixture of Rare Earth Elements to Hydra vulgaris
Source: Toxics. 2024 Dec 12;12(12):904. doi: 10.3390/toxics12120904 (PMC11728654; doi:10.3390/toxics12120904)
Supplement: Supplementary file 1 [file toxics-12-00904-s001.zip › toxics-3367831-supplementary.pdf]

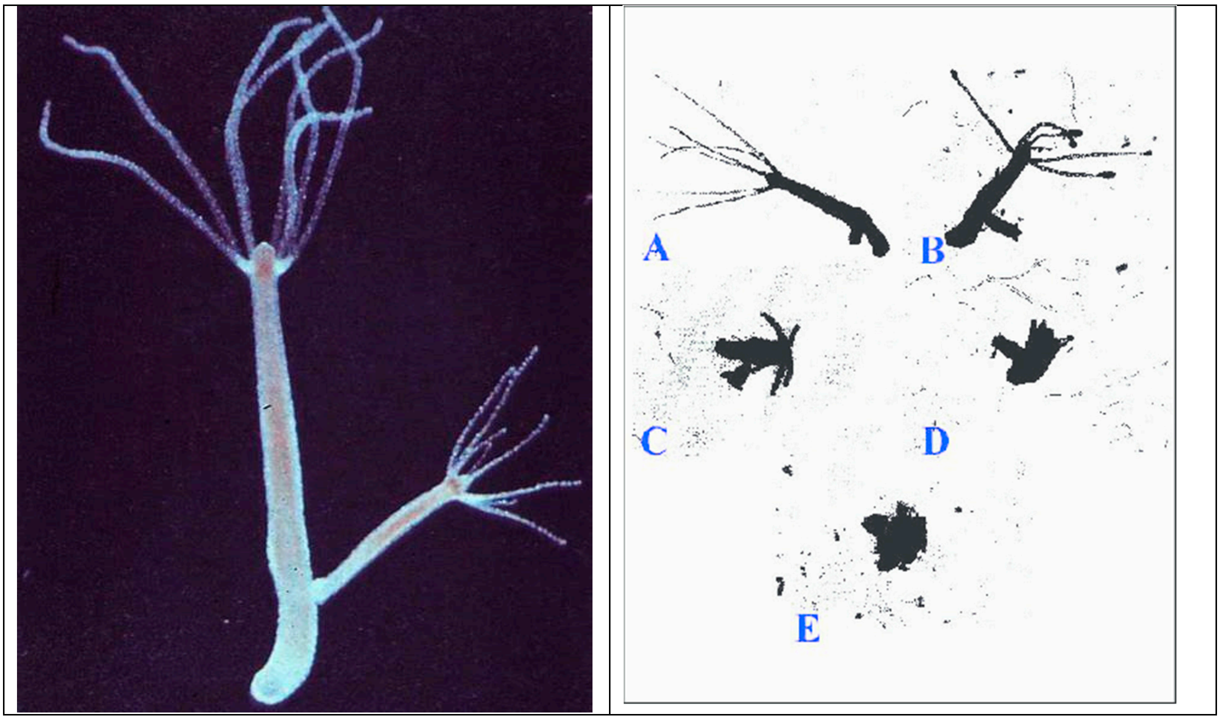

Figure S1. Characteristic morphological changes in Hydra during toxicity.
Normal A, tentacle budding B, tentacle contraction C; tulip stage D and disintegrated E. Sublethal changes are tentacles shortening and/or budding (B and C) and the following are considered irreversible and lethal.
